# Supplementary material for: MetaRibo-Seq measures translation in microbiomes
Source: Nat Commun. 2020 Jun 29;11:3268. doi: 10.1038/s41467-020-17081-z (PMC7324362; doi:10.1038/s41467-020-17081-z)
Supplement: Supplementary file 10 — Supplementary Data 7 [file 41467_2020_17081_MOESM10_ESM.zip › File2/Confidence_High_Taxonomy/260054_out.krona.html]

Javascript must be enabled to view this page.

members
magnitude
magnitudeUnassigned
count
unassigned
taxon
rank

260054\_out

105

105
superkingdom
2

105
phylum
976

class
200643
105

105
171549
order

2
family
171550

2
239759
genus

1
species
28117

SRS062701\_contig\_number\_7213

species

SRS015960\_contig\_number\_11181
1263036
1

family
171551
3

genus
836
3

species

SRS015190\_contig\_number\_2779SRS019445\_contig\_number\_33165SRS043411\_contig\_number\_3516
658663
3

815
family
17

816

SRS148196\_contig\_number\_39768
17
genus
1

1
species
1263038

SRS043701\_contig\_number\_15005

species
818

SRS045195\_contig\_number\_2233SRS050299\_contig\_number\_3900SRS078242\_contig\_number\_11972
3

820

SRS018541\_contig\_number\_12643SRS065397\_contig\_number\_12686
species
2

species
1739517

SRS143523\_contig\_number\_20748
1

162156

SRS023583\_contig\_number\_10686
species
1

469590

SRS148874\_contig\_number\_4432
species
1

species

SRS011239\_contig\_number\_3452SRS050752\_contig\_number\_25137SRS053398\_contig\_number\_167
821
3

species

SRS016056\_contig\_number\_318
46506
1

246787

SRS078419\_contig\_number\_12660
species
1

469592

SRS1041036\_contig\_number\_1318SRS1041138\_contig\_number\_7300
species
2

83
family
2005525

375288
genus
83

79
species

SRS011134\_contig\_number\_46750SRS013098\_contig\_number\_28903SRS013800\_contig\_number\_25192SRS014235\_contig\_number\_31315SRS014313\_contig\_number\_1690SRS014412\_contig\_number\_17792SRS014459\_contig\_number\_38651SRS015065\_contig\_number\_25481SRS015217\_contig\_number\_11070SRS015264\_contig\_number\_11281SRS015369\_contig\_number\_1388SRS015579\_contig\_number\_20900SRS015890\_contig\_number\_12521SRS016989\_contig\_number\_12941SRS017191\_contig\_number\_10484SRS017521\_contig\_number\_37490SRS017701\_contig\_number\_6921SRS018656\_contig\_number\_1291SRS018836\_contig\_number\_25655SRS018984\_contig\_number\_10257SRS019178\_contig\_number\_4314SRS019267\_contig\_number\_6645SRS019397\_contig\_number\_9155SRS019601\_contig\_number\_22571SRS019685\_contig\_number\_35049SRS022609\_contig\_number\_40453SRS024132\_contig\_number\_39162SRS024435\_contig\_number\_37313SRS043768\_contig\_number\_30409SRS046712\_contig\_number\_2492SRS048060\_contig\_number\_10371SRS048164\_contig\_number\_3708SRS049773\_contig\_number\_31705SRS049900\_contig\_number\_13357SRS050422\_contig\_number\_29578SRS051610\_contig\_number\_8901SRS051882\_contig\_number\_11804SRS052027\_contig\_number\_149SRS052078\_contig\_number\_2046SRS053649\_contig\_number\_10756SRS054590\_contig\_number\_1350SRS056259\_contig\_number\_13403SRS058723\_contig\_number\_5222SRS064645\_contig\_number\_1435SRS064973\_contig\_number\_1913SRS065504\_contig\_number\_3874SRS075398\_contig\_number\_1016SRS076804\_contig\_number\_17675SRS077335\_contig\_number\_5069SRS097920\_contig\_number\_14504SRS098061\_contig\_number\_8777SRS098655\_contig\_number\_9372SRS100021\_contig\_number\_5664SRS104036\_contig\_number\_3308SRS1041091\_contig\_number\_10762SRS1041095\_contig\_number\_5422SRS1041142\_contig\_number\_3371SRS1041147\_contig\_number\_704SRS1055049\_contig\_number\_14816SRS140492\_contig\_number\_contig-100\_1293.186601SRS142712\_contig\_number\_4084SRS143148\_contig\_number\_24219SRS143342\_contig\_number\_2828SRS143372\_contig\_number\_9495SRS143895\_contig\_number\_41323SRS144183\_contig\_number\_40678SRS145497\_contig\_number\_12245SRS146764\_contig\_number\_18052SRS147272\_contig\_number\_1566SRS147377\_contig\_number\_2937SRS147614\_contig\_number\_4575SRS148511\_contig\_number\_6593SRS148784\_contig\_number\_14947SRS893259\_contig\_number\_3226SRS893285\_contig\_number\_1794SRS893288\_contig\_number\_3778SRS893341\_contig\_number\_8761SRS893382\_contig\_number\_1187SRS971275\_contig\_number\_24432
823

2
species
1947048

SRS045826\_contig\_number\_13041SRS144506\_contig\_number\_4760

1

SRS018888\_contig\_number\_7497
46503
species

1
species

SRS016095\_contig\_number\_29879
2293115
